# Annexin B12 Trimer Formation is Governed by a Network of Protein-Protein and Protein-Lipid Interactions

**DOI:** 10.1038/s41598-020-62343-x

**Published:** 2020-03-24

**Authors:** Meixin Tao, J. Mario Isas, Ralf Langen

**Affiliations:** Department of Neuroscience and Physiology, Department of Biochemistry and Molecular Medicine, Zilkha Neurogenetic Institute, Keck School of Medicine, University of Southern California, Los Angeles, CA 90033 USA

**Keywords:** Biophysics, Structural biology

## Abstract

Membrane protein oligomerization mediates a wide range of biological events including signal transduction, viral infection and membrane curvature induction. However, the relative contributions of protein-protein and protein-membrane interactions to protein oligomerization remain poorly understood. Here, we used the Ca^2+^-dependent membrane-binding protein ANXB12 as a model system to determine the relative contributions of protein-protein and protein-membrane interactions toward trimer formation. Using an EPR-based detection method, we find that some protein-protein interactions are essential for trimer formation. Surprisingly, these interactions are largely hydrophobic, and they do not include the previously identified salt bridges, which are less important. Interfering with membrane interaction by mutating selected Ca^2+^-ligands or by introducing Lys residues in the membrane-binding loops had variable, strongly position-dependent effects on trimer formation. The strongest effect was observed for the E226Q/E105Q mutant, which almost fully abolished trimer formation without preventing membrane interaction. These results indicate that lipids engage in specific, trimer-stabilizing interactions that go beyond simply providing a concentration-enhancing surface. The finding that protein-membrane interactions are just as important as protein-protein interactions in ANXB12 trimer formation raises the possibility that the formation of specific lipid contacts could be a more widely used driving force for membrane-mediated oligomerization of proteins in general.

## Introduction

A plethora of biological events, including signal transduction^1–4^, viral infection^5,6^ and membrane curvature induction^7,8^ rely on the ability of peripheral membrane proteins to oligomerize on membranes. In addition to physiologically relevant oligomerization of peripheral membrane proteins, it has also been suggested that membranes can promote the pathological oligomerization of amyloidogenic proteins^9–11^. Oligomerization requires specific protein-protein contacts to be made in a process generally known to be facilitated by shape complementarity and hydrophobic as well as electrostatic interactions at the protein contact interface^12–14^. Membranes could further facilitate the formation of such protein-protein contacts in a variety of ways. First of all, membranes provide a common interaction surface that increases the local protein concentration and that reduces the dimensionality of protein diffusion from 3D to 2D. Both mechanisms have been frequently invoked in order to explain the membrane-mediated aggregation of amyloidogenic proteins^9,15–18^. Beyond such a simple surface effect, membranes could also act by more specific interactions, where membranes become a more integral part of the complexes or where modulation of membrane thickness promotes oligomerization. Support has been obtained for transmembrane proteins^19,20^ and computational studies suggest that similar mechanisms could also apply to peripheral membrane proteins^21,22^. However, little experimental support has been obtained thus far in favor of the notion that thickness deformation or other lipid-binding related factors can specifically promote the oligomerization of peripheral membrane proteins.

Here we sought to develop an experimental model system that makes it possible to dissect the effects of protein-protein and protein-lipid interaction on the oligomerization of peripheral membrane proteins. Toward this end, we used trimer-forming annexin B12 (ANXB12) as a model system. ANXB12 belongs to the annexin superfamily of proteins, which are characterized by reversible, Ca^2+^-dependent binding to negatively charged membranes that typically contain phosphatidylserine^23–25^. Annexins have a number of membrane-related functions, including membrane trafficking^26,27^, membrane repair^28–30^, calcium signaling^31,32^ and ion channel formation^33–35^.

The annexin superfamily of proteins is highly conserved and annexins are typically monomeric in solution, yet some annexins form trimers upon Ca^2+^-mediated membrane binding, while others do not. Examples of trimer-forming annexins are annexin A5 (ANXA5) and ANXB12. *In vitro* experiments found that trimer formation is relatively rapid (sub-second) and trimers can subsequently further assemble into 2D networks on a minute to hour time scale^36^. In the case of ANXA5, it has been suggested that these membrane-mediated assemblies are related to annexin’s role in anticoagulation^37^ as well as the repair patch formation in cell membrane wound healing^38^. Interestingly, human ANXA5 and hydra ANXB12 can also form heterotrimers^39^, suggesting that different trimer-forming annexins share a common mechanism of trimer assembly that has remained conserved throughout evolution. In contrast, annexin A1 (ANXA1) and annexin A2 (ANXA2) are non-trimer forming. Trimer and non-trimer forming annexins also vary in terms of their membrane binding ability. Specifically, the trimer-forming annexins bind membranes with much higher calcium stoichiometry (~11 Ca^2+^-ions in the case of ANXB12 as opposed to ~2 in the case of ANXA2^39^). Trimer-forming annexins also strongly inhibit inter-leaflet flip-flop of lipid molecules, while the non-trimer forming annexins are less effective. Furthermore, non-trimer forming annexins can bind to liquid and gel phase membranes, yet trimer-forming annexins only bind to liquid phase membranes^39^. These findings show that trimer and non-trimer forming annexins have distinctively different modes of membrane binding, but it remains unclear whether these differences are directly related to whether the various annexins form trimers or not.

**Figure 1:**
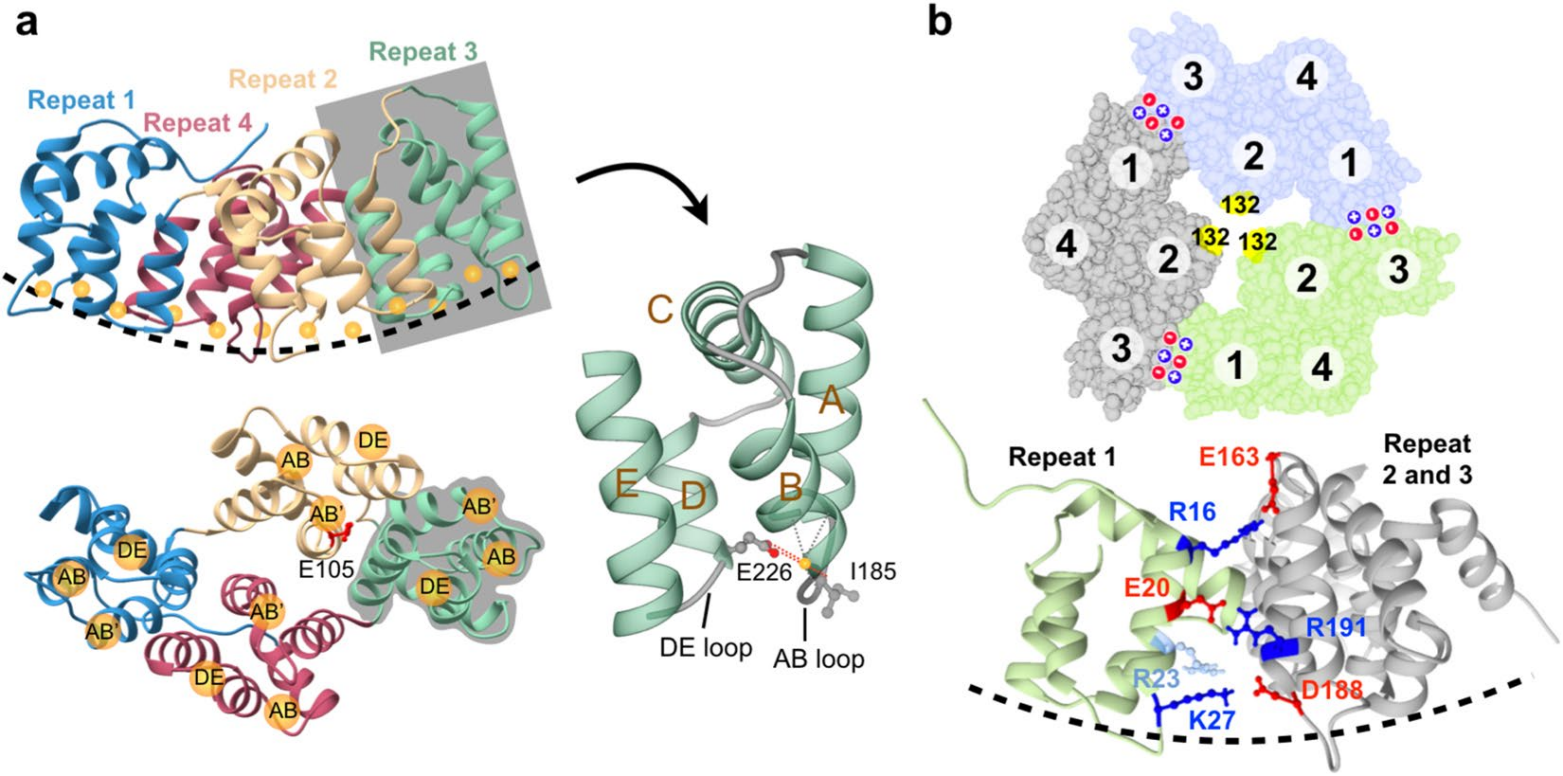
ANXB12 monomer and trimer structures showing Ca^2+^- and lipid-binding sites and inter subunit salt bridges. **(a)** The monomeric subunit of ANXB12 contains four repeats shown in different colors: repeat 1, blue; repeat 2, yellow; repeat 3, green; and repeat 4, red. The membrane-facing surface of ANXB12 is curved (dashed line, top panel). Ca^2+^-ions are shown as orange spheres. The twelve Ca^2+^- and lipid-binding sites belong to three categories: AB, AB′ and DE (bottom panel). The side chain of AB′ Ca^2+^- ligand E105 in repeat 2 is red. Each repeat is composed of five helices (A to E) as illustrated with the zoomed-in depiction of repeat 3 (right). **(b)** The top panel shows the crystal structure of the ANXB12 trimer with subunits highlighted in green, light blue and grey. Residue 132, which is used for the EPR-based detection of trimer formation, is located near the 3-fold symmetry axis and is shown in yellow. The location of positively and negatively charged salt bridge residues are indicated by blue + and red - symbols. The bottom panel zooms in on the salt-bridge interactions which are shown using a side view for one of the trimer interfaces. Residue R23 (which could potentially pair with D188 upon loss of K27) is colored in light blue. All illustrations were created using the ANXB12 crystal structure (PDB code: 1aei).

ANXB12 has four repeats, each composed of a four helical bundle (helices A, B, D, E) and a fifth helix (helix C), which runs perpendicular to the bundle (Fig. 1a). Membrane interaction is mediated by the loop regions between helices A and B (AB loop) and helices D and E (DE loop). These loop regions, which are located on the convex surface of ANXB12 (dashed line in Fig. 1a), harbor up to three Ca^2+^-binding sites (labelled AB, AB′ and DE in Fig. 1a) in each of the four repeats, totalling twelve potential binding sites per protein. The Ca^2+^-binding sites promote membrane binding using a calcium bridging mechanism by which the lipids and the protein jointly coordinate Ca^2+^-ions at the protein-membrane interface. The primary Ca^2+^ and membrane binding sites are the AB loops, which make backbone carbonyl contacts to the Ca^2+^-ion (Fig. 1a). In addition, a key interaction comes from the side chain carboxylate from a Glu or Asp residue in the DE loop within the same repeat (Supplemental Fig. S1). Selective disruption of a subset of AB binding sites has been shown to affect lattice formation, but not membrane binding^40^. However, it is not yet known whether the mutations also affect trimer formation. Trimer formation is very rapid (in milliseconds) and occurs independently of lattice formation, which requires minutes to hours^36^. Loss of trimer formation under these conditions would only be expected, if protein-protein interactions alone are insufficient to induce trimer formation. According to crystal structures of ANXB12 and ANXA5 trimers, a small number of amino acids are found at the contact surface between subunits. The most recognized contacts in the literature^41^ have been the salt bridges between individual subunits (shown for ANXB12 in Fig. 1b). Disruption of these salt bridges in ANXA5 has also been shown to reduce lattice formation^38^. It is therefore possible that the salt bridges are the key factors in governing trimer formation. However, the direct effect of the salt bridges on membrane-mediated trimer formation has not yet been determined and it is not clear whether other protein-protein contacts exist to have significant contributions to trimer formation.

One of the central goals of the present study was therefore to obtain quantitative experimental data for the effects of protein-protein and protein-lipid interaction on ANXB12 trimer formation. Surprisingly, we found that the impact of salt bridges on trimer formation, though detectable, was relatively small. In contrast, mutations of other residues at subunit interface, had a much more pronounced effect on trimer formation. These residues engaged in a combination of hydrophobic and electrostatic interactions. While protein-protein interactions were important, they were not sufficient for inducing trimer formation as disruption of some Ca^2+^- and lipid-binding sites also strongly inhibited trimer formation on membranes. Collectively, our data suggest that trimer formation of ANXB12 is simultaneously driven by a network of protein-protein and protein-membrane interactions.

## Results

**Figure 2:**
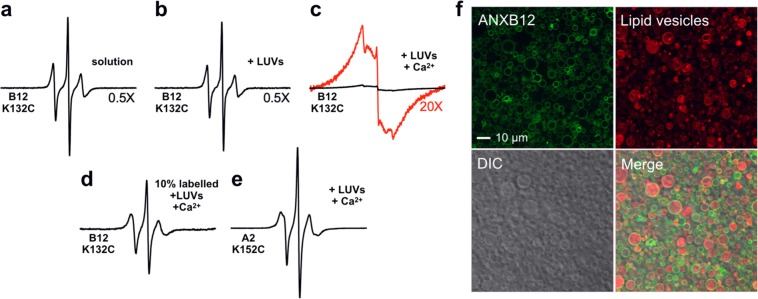
EPR and fluorescence-based detection of ANXB12 membrane binding and trimer formation. All black EPR spectra in panels (**a–e**) are shown normalized to the same number of spins. **(a)** EPR spectrum of ANXB12 132R1 was recorded in solution (20 mM HEPES, 100 mM NaCl, pH 7.4). **(b)** In the absence of added Ca^2+^, the addition of 1000 nm LUV (POPS/POPC molar ratio 2 to 1) at a protein-to-lipid molar ratio of 1:450 does not cause membrane binding and results in spectrum analogous to that in (**a**). **(c)** Membrane binding was induced using the same condition as in (**b**), but in the presence of 1 mM CaCl_2_. The spectrum is of much lower amplitude and significantly broadened. To better visualize the line shapes, the red spectrum shows the same spectrum at 20X magnification. **(d)** The EPR spectrum of membrane-bound 132R1 as in (**c**) but diluted with 90% unlabelled protein (ANXB12 Cysless). This spin dilution leads to much sharper lines and an increase in EPR central line amplitude relative to the spectrum in (**c**). **(e)** The EPR spectrum of the 152R1 derivative of ANXA2 in membrane-bound form (same condition as in (**c**)). 152R1 in ANXA2 is homologous to 132R1 in ANXB12. ANXA2 is non-trimer forming and the EPR spectrum of its 152R1 derivative does not exhibit spin-spin interaction. **(f)** Fluorescent images showing ANXB12 (green) bound to vesicles (red). ANXB12 was labelled using Alexa Fluor 488 and mixed with 0.2% rhodamine DOPE-labelled, sucrose-filled vesicles (POPS/POPC 2 to 1, red) in buffer (20 mM HEPES, 100 mM NaCl, 1 mM CaCl_2_, pH 7.4).

To determine how protein-protein and protein-lipid interactions affect Ca^2+^- and membrane-dependent trimer formation, we specifically disrupted these interactions in a total of 22 mutants. A list of all mutants can be found in Table 1 and details of mutated residues are summarized in Supplemental Fig. S1. All mutants were purified using reversible, Ca^2+^-dependent membrane binding, indicating that they were functional with respect to membrane binding. The extent of trimer formation was quantified using a previously established, EPR-based method. This method detected trimer formation using spin-spin interaction that occurs as three spin labels at residue 132 (132R1) come into close proximity^36^ (Fig. 1b, yellow). Residue 132 is located in a loop region, where it is highly exposed and accessible, further ensuring high labelling efficiencies. In fact, essentially quantitative labelling has previously been demonstrated for this residue^36^. In solution, ANXB12 has been shown to be monomeric^36,42^, and the EPR spectrum of 132R1 gave rise to three sharp lines, characteristic of dynamic spin label motion without significant spin-spin interaction (Fig. 2a). No significant EPR spectral changes were observed when large unilamellar vesicles (LUV) were added to 132R1 in the absence of additional Ca^2+^ (Fig. 2b), consistent with prior reports that no membrane binding occurs under these conditions^36,42^. However, membrane binding was induced by the addition of Ca^2+^. According to fluorescence microscopy the vesicles remained largely intact under these conditions (Fig. 2f) without being remodelled into smaller vesicles, as sometimes observed with other proteins^43^. As previously described, these conditions led to trimer formation and resulted in a distinctively different EPR spectrum with significant amplitude drop and broad lines indicative of strong spin-spin interaction (Fig. 2c). The effect of spin-spin interaction was alleviated by mixing labelled protein with 90% unlabelled ANXB12 (B12 Cysless), which gave rise to sharp spectral lines of significantly increased signal amplitude (Fig. 2d). A similar EPR spectrum with sharp lines and high intensity was also obtained for fully R1-labelled, membrane-bound ANXA2 spin-labelled at residue 152 (152R1), a position that is equivalent to 132R1 in ANXB12 (Fig. 2e). Since ANXA2 does not undergo membrane-mediated trimerization, the 152R1 spectrum does not exhibit spin-spin interaction. Collectively, these data confirm prior reports that spin-spin interaction can be used to detect ANXB12 trimer formation and that loss of trimer formation causes loss of spin-spin interaction^36,39^.

**Table 1:** Summary of ANXB12 mutants in the study.

Category	Mutations	Acronym
Disrupted Protein-Protein Contacts	R16A/R191A/K27A/R23A	SB-A
	R16E/R191E/K27E/R23E	SB-E
	F59A/Q148A/R149A	IF-A
	R16A/R191A/K27A/R23A/F59A/Q148A/R149A	SBIF-A
Disrupted Protein-Lipid Contacts	E70Q	AB1
	E142Q	AB2
	E226Q	AB3
	D301N	AB4
	E70Q/E142Q	AB12
	E70Q/E226Q	AB13
	E70Q/D301N	AB14
	E142Q/E226Q	AB23
	E142Q/D301N	AB24
	E226Q/D301N	AB34
	E70Q/E142Q/D301N	AB124
	E142Q/E105Q	AB2AB′2
	E226Q/E105Q	AB3AB′2
	I29K/L101K/I185K/L260K	K1234
	E70Q/I29K	AB1K1
	E142Q/I101K	AB2K2
	E226Q/I185K	AB3K3
	D301N/L260K	AB4K4

In addition to the mutations listed, all ANXB12 derivatives listed in the table are also R1 spin-labelled at residue 132 to allow EPR-based detection of trimer formation. The 22 mutants are divided into two main categories where protein-protein or protein-lipid contacts were disrupted. The right column specifies the acronym of each mutant as it is referred to in the paper.

**Figure 3:**
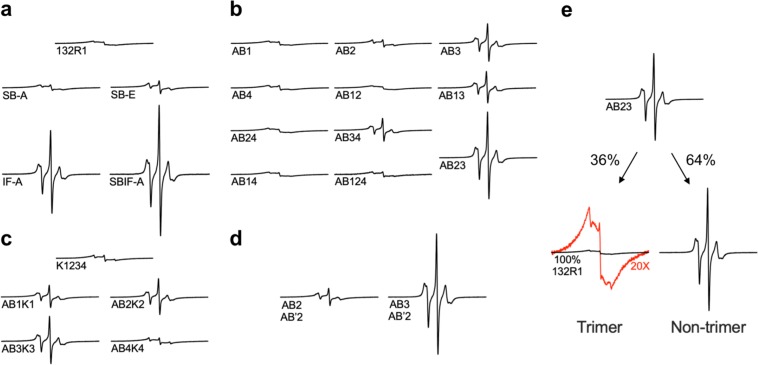
EPR spectra of ANXB12 derivatives with mutations designed to disrupt protein-protein or protein-membrane interactions. All mutants contain a spin label at position 132 to monitor trimer formation. Additional mutations are made to disrupt protein-protein **(a)** or protein-lipid interactions **(b–d)**. The acronyms for all mutants are described in Table 1. The EPR spectra of ANXB12 mutants with disrupted Ca^2+^-ligands in their AB Ca^2+^-binding sites are given in **(b)**. Lysine substitutions at AB Ca^2+^-binding sites either alone or in combination with Ca^2+^-ligand disruptions are presented in **(c)**. EPR spectra of mutants in which selected AB and AB′ Ca^2+^-ligands are simultaneously mutated are shown in **(d)**. All spectra have varying amounts of two spectral components arising from a trimer and a non-trimer state. Deconvolution of the respective spectral components is shown for the AB23 mutant in **(e)**. The trimer component is of very low amplitude and to better visualize its line shape, this spectrum is also plotted at 20X magnification (red). All spectra shown in black were normalized to the same number of spins throughout the figure. This representation illustrates the different amplitudes but makes some of the line shapes more difficult to see. The spectra are therefore replotted at uniform amplitude in Supplemental Fig. S2.

Using the 132R1-based readout, we next determined how trimer formation was affected by mutations at the protein-protein or protein-membrane interface. The membrane-bound state of each annexin mutant was obtained by mixing protein with vesicles (1:450 molar ratio) in the presence of 1 mM Ca^2+^. These conditions were chosen to ensure membrane binding in all cases. The membrane-bound ANXB12 mutants were then concentrated by centrifugation. All mutants pelleted nearly quantitatively with negligible EPR signals remaining in the supernatant. The ability to bind to membranes in all cases was consistent with the fact that all proteins could be purified using reversible, Ca^2+^-dependent membrane binding. As shown in Fig. 3a–d, the amplitude and shape of the EPR spectra of all derivatives varied widely. The differences in amplitude were not due to different concentrations, as all spectra were normalized to the same number of spins. Rather spectral subtractions showed that all spectra were composed of varying ratios of two very different spectral components, illustrated with AB23 mutant in Fig. 3e. One of these spectral components corresponds to the broad, spin-spin interaction containing 132R1 spectrum (Fig. 2c), which is indicative of trimer formation. The other spectral component had narrow lines and was of high amplitude. This spectrum was different from that of ANXB12 132R1 in solution (Fig. 2a) and therefore could not be attributed to unbound protein. Rather, this spectrum was similar to those in Fig. 2d,e and indicated the formation of a membrane-bound state that lacked spin-spin interaction and that no longer formed the trimer. Due to the drastically different amplitudes of the spectral components, it was possible to estimate percent trimer formation from the respective amplitudes, as larger amplitudes indicated less trimer formation. In the following sections, we therefore quantified the spectral amplitudes in triplicates and converted them into percent trimer formation (see Methods).

### Some protein-protein interactions, but not the salt bridges, have a strong effect on ANXB12 trimer formation

**Figure 4:**
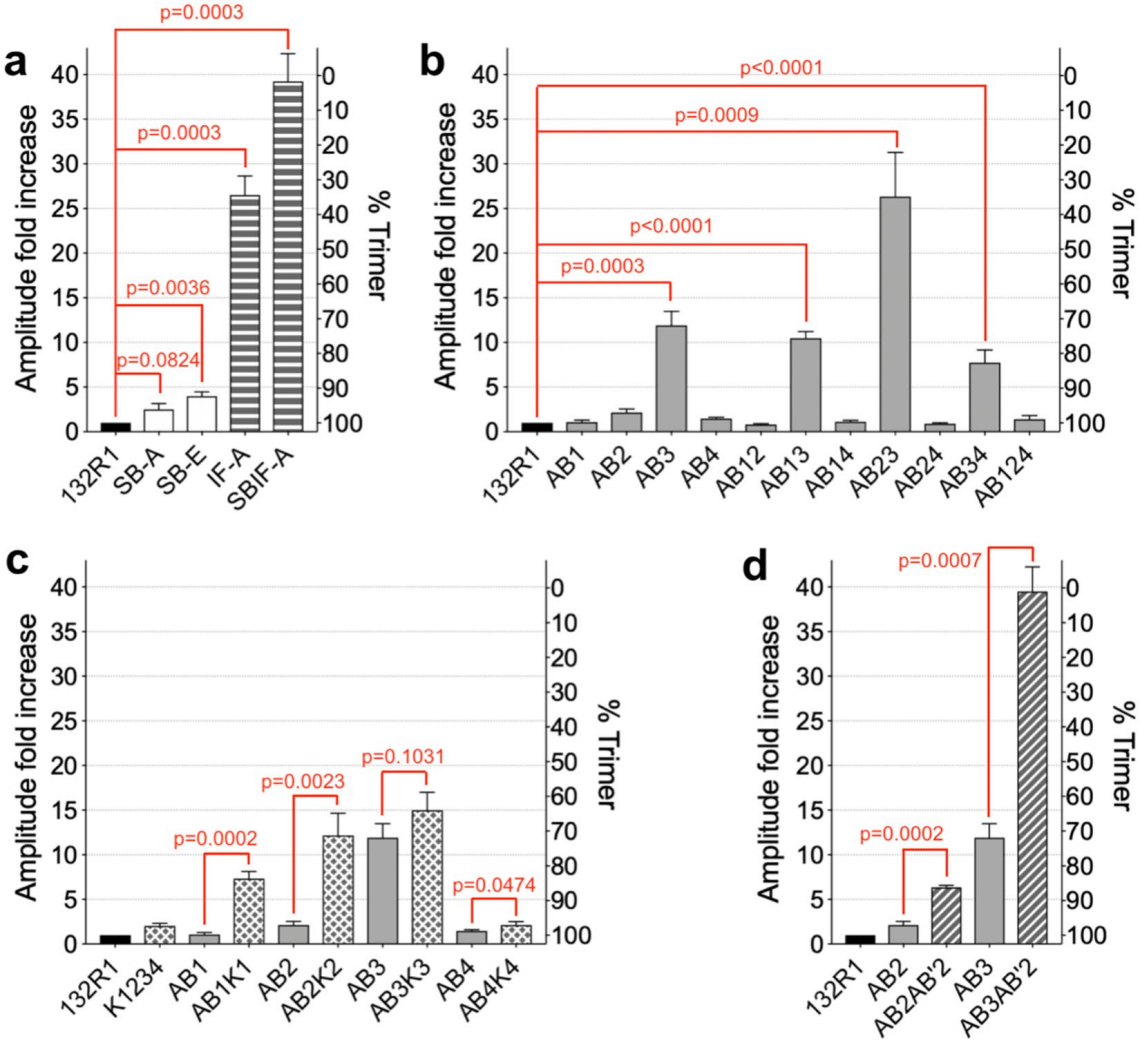
ANXB12 trimer formation estimated from EPR amplitudes. The amplitudes of the EPR spectra for the various mutants are shown as fold increase relative to the amplitude of 132R1. All amplitudes are those of spin normalized EPR spectra obtained in triplicates. The second y-axis shows the percentage of trimer formation, which is inversely proportional to the relative amplitude. The mutants are grouped into disruption of (**a**) protein-protein interactions, (**b**) AB Ca^2+^-binding ligands, (**c**) lysine introduction into the AB loops and (**d**) simultaneous disruption of ligands in the AB and AB′ Ca^2+^-binding sites. The mutant names are explained in Table 1. The bars represent average values of independent repeats (n = 3) with standard deviation. Two-tailed unpaired t-tests were performed for selected mutant pairs and P-values are shown (red). Data analysis was performed in GraphPad Prism 8.0.

To test the effect of protein-protein interactions, we first mutagenized three pairs of salt bridges (R16-E163, E20-R191, K27 (or R23)-D188), which are the primary interaction sites between the monomers (Fig. 1b). It is worth noting that K27 as well as R23 could potentially salt bridge with D188, as has been suggested in case of the homologous residues in ANXA5^41^. Both residues were therefore mutated. Two different types of salt bridge-disrupting mutants were made: SB-A and SB-E. For the SB-A mutant, the positively charged salt bridge residues (Supplemental Fig. S1, blue) were replaced by alanine, thereby leaving the negatively charged residues without counterion. For the SB-E mutant, the positively charged residues were replaced with negatively charged glutamic acids. This mutant was designed to be more severe as it replaced the three salt bridges with negatively charged residues that would be expected to repel each other. When compared to the 132R1 reference spectrum (Fig. 3a, magnified in Supplemental Fig. S2), increased amounts of sharp lines could be observed for SB-A and for SB-E this effect was more pronounced. Nonetheless, all spectra were still of relatively low amplitude and contained clear evidence of abundant trimer formation. In fact, based on the EPR spectra we estimated that trimer formation was ~96% and 92% for SB-A and SB-E, respectively (Fig. 4a). This result was particularly surprising in the case of the SB-E mutant, where charge repulsion might have been expected to result in stronger effects. These data suggested that the salt bridges alone were not the primary drivers of trimer formation, rather additional and possibly stronger driving forces had to be present.

To test the effects of other protein-protein interactions, we mutagenized additional residues located at the subunit interface (F59, Q148 and R149, Supplemental Fig. S2, orange) into alanines, resulting in the interface alanine mutant IF-A. As illustrated in Supplemental Fig. S3, this mutant is disrupted in numerous contacts, most of which were hydrophobic in nature, although electrostatic interactions are also disrupted. In contrast to the salt bridge mutants, this mutant had a much more pronounced effect, resulting in only 35% trimer formation. When combined with the salt bridge alanine mutant (SBIF-A), trimer formation was almost completely abolished (3% trimer). These results indicated that specific protein-protein interactions play an important role in ANXB12 trimer formation, but that a combination of mutations was required to observe strong effects.

### Some Ca^2+^- ligands in AB membrane binding sites significantly contribute to ANXB12 trimer formation on membranes

**Figure 5:**
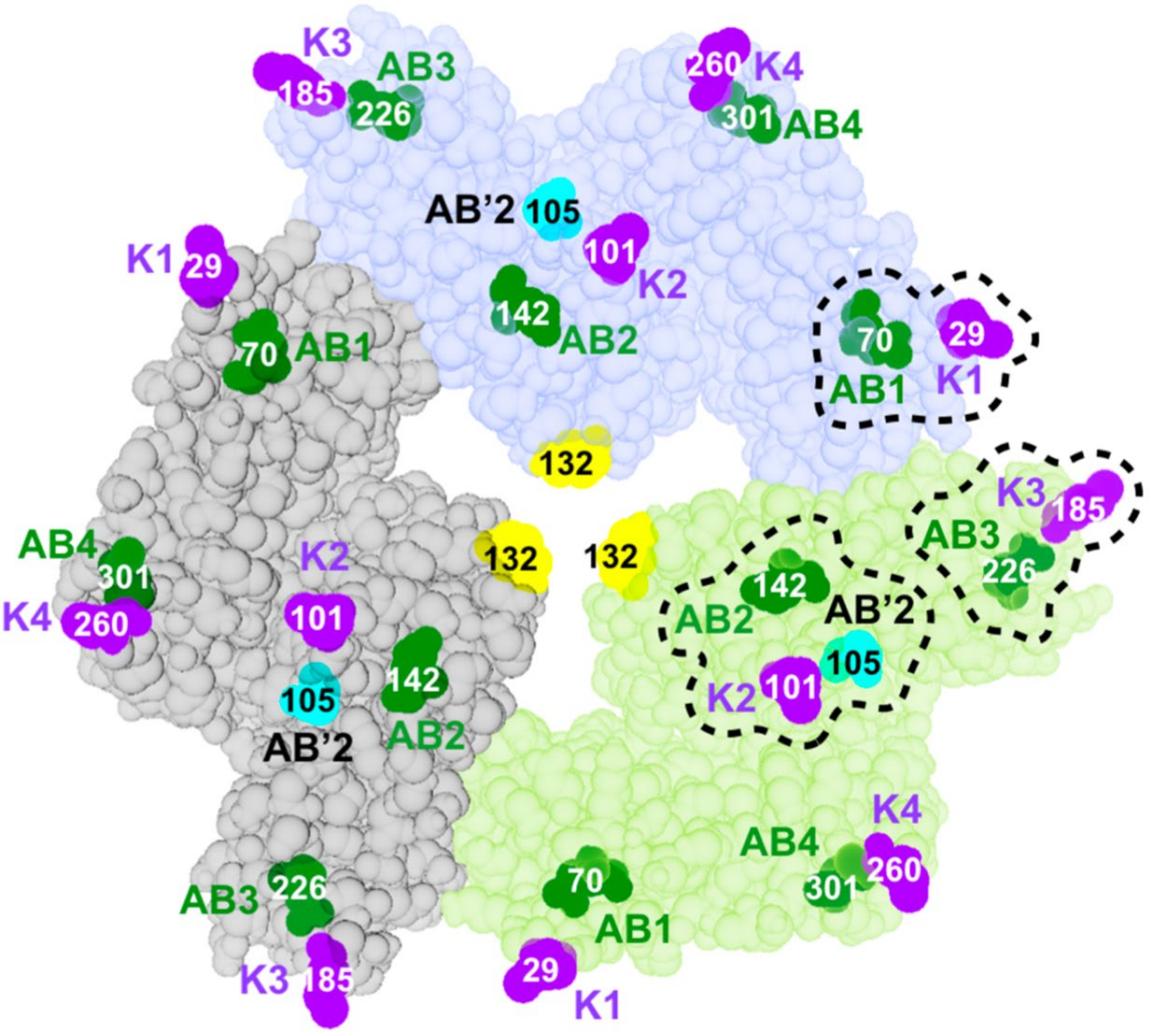
Convex surface of the ANXB12 trimer showing the location of disrupted protein-lipid contacts. Each monomer of the ANXB12 trimer is illustrated with a different color. Mutagenized Glu/Asp ligands in AB Ca^2+^-binding sites are colored in green. The E105 ligand of the repeat 2 AB′ Ca^2+^-binding site is colored in cyan. Locations where lysine residues were introduced are purple. The illustration was created from the ANXB12 crystal structure (PDB code: 1aei). Most of the protein-membrane contacts that are critical to trimer formation of ANXB12 (repeat 1, 2 and 3, circled with dashed lines) are located in close proximity to the subunit interface within the trimer.

We next examined the contribution of protein-lipid interaction to ANXB12 trimer formation. As described above, AB Ca^2+^-binding sites play a major role in ANXB12 membrane interaction (Fig. 1a). To test the influence of these binding sites on trimer formation, we mutagenized the conserved Glu/Asp residues (see Supplemental Fig. S1 and Fig. 5, green) within the DE loops to their respective amidated forms (i.e. E into Q or D into N). Although the mutated residues reside in the DE loops, they coordinate Ca^2+^ in the AB loops. We therefore referred to these mutants as AB1, AB2, AB3 and AB4, where the number indicated the repeat in which the mutations were made. As shown with the EPR spectra in Fig. 3b and the analysis in Fig. 4b, AB1, AB2 and AB4 had only a very modest effect, while AB3 caused more significant disruption of trimer formation (72% trimer). Surprisingly, the disruption of trimer formation caused by a single ligand (AB3) was more pronounced than for either of the salt bridges mutants.

To further investigate how combinations of Ca^2+^-binding site mutants affected trimer formation, we generated six additional proteins that had mutations in two AB binding sites in different repeats: AB12, AB13, AB14, AB23, AB24 and AB34 (again numbers indicate repeat in which mutations were made). The impact of each double mutant on trimer formation is shown with the EPR spectra in Fig. 3b and the analysis in Fig. 4b. All double mutants containing a mutation in repeat 3 (AB23, AB13, and AB34) showed a significant drop in trimer formation. All other double mutants had only minor effects. In fact, even the triple mutant, where all ligands in repeats 1, 2 and 4 were mutated (AB124) had little effect on trimer formation (Fig. 4b). While ligand mutation in repeat 3 was clearly the dominant event, differences were observed depending on where the second mutation was placed. That is, the AB23 mutant had a very low degree of trimer formation (36% trimer), much lower than that of the individual AB2 (97% trimer) and AB3 (72% trimer) mutations alone. Thus, there was strong synergy when these two ligands were mutated simultaneously. The second highest disruption was seen for the AB13 (76% trimer) mutation, which was similar to that of AB3 alone. The lowest disruption of trimer formation for the AB3-containing mutants was observed for AB34 (83% trimer).

### ANXA2-mimicking Lys mutations at the tip of AB binding loops cause a modest reduction of trimer formation

Sequence alignment between trimer-forming ANXB12 and non-trimer forming ANXA2 showed that ANXA2 has lysine substitutions for the membrane-facing Ile/Leu residues within the AB loop (Supplemental Fig. S1 and Fig. 5, purple). According to a computational simulation study, positively charged lysine residues on the membrane-facing convex surface can facilitate ANXA2-membrane interaction via electrostatic interactions with negatively charged lipids^44^. Whether these interactions also affect trimer formation, however, is unknown. To test whether ANXA2-mimicking lysine substitutions could destabilize trimer formation of ANXB12, we generated another five mutants: K1234 (simultaneously substituting all four Ile/Leu residues into lysines) and AB1K1, AB2K2, AB3K3, AB4K4 (mutating the Glu/Asp residue and the Ile/Leu residue within each repeat). Again, the numbers refer to the repeat in which the mutations were introduced. The introduction of the four lysines alone reduced trimer formation to 97% trimer. This effect was relatively small and comparable to that of the SB-A salt bridge mutant. Thus, Lys mutations alone are not major disruptors of trimer formation and the corresponding Lys residues are likely not responsible by themselves for the lack of trimer formation in ANXA2. Nonetheless, the introduction of lysines also further potentiated the effects of the ligand mutations. The AB3 mutation alone already had significant effects on trimer formation (72% trimer) and the AB3K3 mutant still further enhanced this effect (64% trimer). Moreover, while the single ligand mutations in the other repeats had no significant trimer disrupting effects, the additional lysine mutations caused robust trimer disruption for AB1K1, AB2K2 and AB4K4 (Figs. 3c and 4c). Among these three mutants, the strongest effect was seen for AB2K2 and AB1K1 (72% and 84% trimer respectively), while a much more modest effect was observed for AB4K4 (97% trimer). Interestingly, these data give the same order of importance for the repeats as the ligand double mutants mentioned above with the importance decreasing in the following manner: repeat 3≫ repeat 2> repeat 1≫ repeat 4.

### The AB′ ligand E105 is important for trimer formation

The Ca^2+^- and membrane-binding mutations thus far only involved the AB Ca^2+^-binding sites. However, other binding sites could also contribute to trimer formation. To test this notion, we focused on E105 (Fig. 1a, red), a ligand for the AB′ Ca^2+^-binding site in repeat 2 (Supplemental Fig. S1 and Fig. 5, cyan). It has previously been suggested that AB′ Ca^2+^-binding sites contribute to lipid binding by specifically coordinating the negatively charged serine moiety of the phosphatidyl serine head group^23^. Prior studies revealed that mutating E105 can cause marked alterations in membrane properties^45^. Therefore, we generated the E105Q in combination with the AB2 or the AB3 mutants described above. The resulting mutants are referred to as AB2AB′2 and AB3AB′2, respectively. As shown in Figs. 3d and 4d, both of these mutants caused significantly more reduction in trimer formation than individual AB mutants alone with the AB3AB′2 almost completely abolishing trimer formation (2% trimer). Thus, AB′ sites like E105 also contributed to trimer stability.

Our prior studies showed that the labelling at position 132 is essentially quantitative^36^. To further ensure that the line shape changes seen here were not a consequence of underlabelling in some cases, we evaluated the labelling efficiencies of mutants which exhibited the strongest line shape changes, including AB3AB′2. As illustrated in Supplemental Fig. S4, all mutants were as fully labelled as the K132C reference mutant. Thus, lack of labelling was not the reason for the line shape changes. Moreover, even if underlabelling of about 10% were present, its spectral effects would be limited, as loss of one spin label in the trimer (statistically most likely scenario^36^) would still leave two spin labels in close proximity and this would also cause strong amplitude reductions (Supplemental Fig. S4d).

## Discussion

The present study investigated the effects of protein-protein and protein-membrane interactions on ANXB12 trimer formation. While selective mutations of only three or four residues at the protein-protein interaction surface had only small effects, a combined mutant in which seven contact residues were substituted (SBIF-A mutant) nearly completely abolished trimer formation. This indicated that protein-protein interactions were essential and that multiple individual contacts were needed to bring about trimer formation. Perhaps more surprisingly, we found that similar trimer disruption could be achieved by mutating only two residues (AB3AB2′) on the membrane binding surface. Protein-membrane interactions are therefore just as important as protein-protein interactions in terms of promoting trimer formation and both interactions are required for trimer formation. By selectively targeting only a subset of up to 12 Ca^2+^- and lipid-binding sites, it was possible to obtain mutants that retained their functional ability to bind to membranes. In other words, all of these mutants still allowed the proteins to be concentrated on the membrane and to experience two-dimensional diffusion. The role of membranes in ANXB12 trimer formation must, therefore, go beyond simply providing a two-dimensional surface. Rather, lipids are likely to engage in some specific interactions that promote trimer formation.

While protein-protein and protein-membrane interactions were clearly essential, not all of the interactions were equally important. For example, we found that the salt bridge mutants did not have a very strong effect on trimer formation (4–8%). This was rather surprising as the salt bridges have typically been considered important for trimer formation. A potential reason for why the salt bridge mutations had a relatively small effect was that the energetic gains from charge interactions in the trimer could have been offset by the unfavorable loss of solvation of the same residues in the monomer. In fact, it has previously been suggested that salt bridges may not always represent strong driving forces for protein-protein interactions^46^. In contrast, the IF-A mutant (F59A/Q148A/R149A) yielded a much bigger effect (65% disruption). This mutation disrupted a variety of interactions, many of which are hydrophobic (Supplemental Fig. S3). However, even this mutant alone only partially disrupted trimer formation and near complete inhibition of trimer formation required the combined SBIF-A mutant, in which seven residues are mutated.

Although the ANXB12 repeats are highly homologous, disrupting the AB Ca^2+^- and lipid-binding sites had very different effects, depending on which repeat was targeted. For example, mutations in repeat 4 had little or no effect on trimer formation, while mutations in repeats 3, 2, and 1 had much more pronounced effects. Interestingly, all of these repeats were located at the subunit interface in the trimer, while repeat 4 was not (Fig. 5). This pattern is consistent with a model, in which specific protein oligomerization above the membrane (trimer formation) is coupled to attractive forces between lipids near the trimer interface. The nature of these interactions is still unclear, but it could be related to two characteristic features of trimer-forming annexins, like ANXB12. One of these features is that trimer-forming annexins strongly reduce lipid and protein movement^47–50^. This immobilization is caused by the formation of a specific lipid network that is accommodated by a complementary spacing between the tightly packed lipids and the membrane binding sites of the assembled trimer^39^. Based on prior studies it is likely that formation of this network is strongly affected by E105 mutation^45^. Moreover, non-trimer forming annexins do not result in lipid immobilization and they do not appear to form a specific lipid network. Another characteristic feature of ANXB12 is that the membrane-binding surface is highly curved and located on the convex side of the protein. Our prior studies^51,52^ revealed a continuous contact between the convex surface and the lipid molecules. By following the convex shape of ANXB12 (see dashed line in Fig. 1a), the membrane is likely to experience thickness deformations. In the trimer, such thickness deformations would be complementary as the most stretched lipid regions are located at the contact surface between monomers in the trimer (Fig. 1). According to recent computational work, the local clustering of stretched or compressed lipid regions is energetically favorable^20,53^. Thus, it is possible that thickness deformations might contribute to trimer formation of ANXA5 and ANXB12, where binding is largely driven by the numerous Ca^2+^- and lipid-binding sites on the convex surface. This effect might be less pronounced for ANXA1 and ANXA2, which have much fewer such binding sites in lieu of potentially more flexible Lys-containing membrane-binding loops. Future studies will have to show the extent to which membrane thickness deformations contribute to trimer formation in annexins.

The finding that protein-lipid contacts can actively control oligomerization of ANXB12 suggests the possibility that similar mechanisms could also apply to other proteins, perhaps even allowing the hetero-oligomerization of different proteins. It has been well established that cellular membranes can be organizing centers that bring a subset of proteins into the same local membrane domain where they promote oligomerization by enhancement of local concentration. By making specific lipids parts of the oligomeric complex it may thus be possible to regulate protein-protein interactions through control of lipid binding interactions and perhaps lipid compositions.

## Methods

### Mutagenesis, expression, purification and spin labelling of ANXB12 mutants

ANXB12 and ANXA2 were expressed using pSE420 plasmids. The ANXB12 cysless mutant, K132C mutant and ANXA2 K152C mutant were generated as described previously^36,39^. All other mutant plasmids were prepared with Q5 Site-Directed Mutagenesis Kit (NEB) using ANXB12 K132C mutant as the template and were sequence confirmed. The mutant plasmids were transformed into DH5α cells (Zymo5α, #T3007) and cells were spread on LB plates (100 μg/ml ampicillin) at 37 °C overnight. A single colony was picked and inoculated into 5 ml TB media with ampicillin (100 μg/ml) for overnight incubation (225 rpm, 37 °C). Cultures were then pelleted and the pellet was resuspended in 5 ml fresh TB media with ampicillin. Of these 500 μl were used to inoculate 50 ml fresh media. This starting culture was incubated for another 3 hours before expanding into two flasks of 500 ml fresh TB media with ampicillin. Protein expression was induced by 1 mM IPTG when O.D._600nm_ reached 1.2, followed by overnight incubation (180 rpm, 18 °C). Bacteria were harvested the next morning by centrifugation (3,500 × g, 4 °C, 20 minutes). Protein purification was based on the reversible Ca^2+^-dependent binding to phosphatidylserine-containing vesicles. Giant unilamellar vesicles were made with the Reeves-Dowben method^54^. Bacterial pellets were incubated in lysis buffer (20 mM HEPES, 100 mM NaCl, 1 mM EDTA, 1 mM EGTA, 1 mM DTT, 0.15 mg/ml egg lysozyme, pH 7.4) for about 30 minutes, then sonicated in the presence of 0.3 μM PMSF, followed by centrifugation (26,000 × g, 4 °C, 40 minutes). The supernatant was transferred and mixed with Reeves-Dowben vesicles in the presence of 5 mM CaCl_2_ followed by centrifugation (26,000 × g, 4 °C, 30 minutes). The pellet was washed by resuspending in washing buffer (20 mM HEPES, 100 mM NaCl, 1 mM CaCl_2_, pH 7.4) followed by centrifugation (18,000 × g, 4 °C, 20 minutes). This process was repeated three times. The pellet was then resuspended in 10 ml elution buffer (20 mM HEPES, 100 mM NaCl, 10 mM EGTA) and again subjected to centrifugation (26,000 × g, 4 °C, 30 minutes). The supernatant was again subjected to centrifugation. The final supernatant was concentrated to 0.5 ml using a 10 kDa Centricon Filter (Millipore #UFC901096). Next, the protein was purified with a Superdex 200 10/300 GL size exclusion column (GE Healthcare) using buffer (20 mM HEPES, 100 mM NaCl, 1 mM EDTA, 1 mM DTT, pH 7.4). The protein eluted in the second peak at ~16 ml elution volume. For MTSL labelling, DTT was first removed using a PD10 column (GE Healthcare). MTSL spin label ((1-Oxyl-2,2,5,5-tetramethyl-∆3-pyrroline-3-methyl) Methanethiosulfonate) (Toronto Research Chemicals, #O875000) was added at> 5:1 (label/protein) molar ratio. According to the labelling kinetics for ANXB12 residue 132, the reaction is complete after 30 minutes incubation at 4 °C. To ensure complete labelling and high label efficiency, the labelling reaction was allowed to proceed overnight. The labelled protein was then passed through a HiTrap Q XL anionic exchange column (GE Healthcare) to wash away any free labels and for final purification. The end product was verified on SDS-PAGE gels as a single band at around 35 kDa, consistent with the molecular weight of annexin monomers. In order to match the osmolarity of lipid vesicles used in the EPR experiments later, buffer exchange (20 mM HEPES, 100 mM NaCl, pH 7.4) was performed for the purified proteins using PD10 columns. Protein concentration was determined by UV absorbance at 280 nm using an extinction coefficient of 14,900 M^−1^ cm^−1^ (ExPASy, ProtParam Tool). To label the K132C reference mutant diamagnetically, (1-Acetoxy-2,2,5,5-tetramethyl-∆3-pyrroline-3-methyl) Methanethiosulfonate (Toronto Research Chemicals, #A167900) was used in replacement of the paramagnetic MTSL.

### Preparation of lipid vesicles

1-palmitoyl-2-oleoyl-sn-glycero-3-phosphatidylcholine (POPC) and 1-palmitoyl-2-oleoyl-sn-glycero-3-[phospho-L-serine] (POPS) were purchased as chloroform solutions from Avanti Polar Lipids, Inc (#850457C and #840034C). Lipids were first mixed (POPS/POPC molar ratio 2 to 1) and then dried under a gentle nitrogen flow to form a thin layer of lipid film and kept in a desiccator overnight at room temperature. The film was later resuspended in buffer (20 mM HEPES, 100 mM NaCl, pH 7.4) to a final concentration of 15 mg/ml. After 10 cycles of freeze-and-thaw, lipid vesicles were extruded through a 1.0 μm Nucleopore Track-Etch Membrane (Whatman, #800319) back and forth for 21 times using glass syringes.

### CW-EPR spectroscopy analysis

For EPR measurements, annexin protein (ANXB12 or ANXA2) were mixed with 600 μg of the extruded vesicles (POPS/POPC molar ratio 2 to 1) in 20 mM HEPES, 100 mM NaCl, pH 7.4 with 1 mM CaCl_2_ (protein-to-lipid molar ratio ~1:450) in a total volume of 1000 ul. The lipid vesicles as well as the membrane-bound annexins were harvested by centrifugation (21,000 × g, room temperature, 20 minutes). After carefully removing the supernatant, the pellet was collected into a borosilicate glass capillary to record EPR spectra using a Bruker EMX spectrophotometer fitted with ER4119HS resonator (scan width 150 G, modulation amplitude 1.5 G). All spectra were normalized to the same number of spins using double integration. The central line amplitudes from the normalized spectra were converted to fold increase over the 100% labelled ANXB12-K132C mutant and plotted. Estimates of trimer formation were then obtained from the spectral amplitudes of the respective normalized spectra assuming a linear combination of the individual spectral components of Fig. 3e.

### Fluorescence microscopy

The ANXB12 K132C mutant was labelled with *Alexa Fluor* 488 C5 Malemide (Invitrogen, #A10254) at 4 °C overnight. Residual free dye was removed by PD10 column (GE Healthcare #17-0851-01). Sucrose-filled (240 mM) Reeves Dowben vesicles composed of POPS/POPC (2 to 1 molar ratio) and labelled with 0.2% rhodamine-DOPE (Avanti Polar Lipids, #810150) were prepared and washed with buffer (20 mM HEPES, 100 mM NaCl, pH 7.4) for 10 minutes at 21,000 × g. Protein and lipid vesicles were mixed at an estimated 1 to 450 protein-to-lipid ratio in the same buffer containing 1 mM CaCl_2_ at room temperature for 20 minutes. The images were acquired using a Zeiss LSM 780 inverted confocal microscope with a 63x oil immersion NA 1.4 objective lens and then processed using Fiji/ImageJ software.

## Data Availability

All data generated or analyzed during this study are included in this published article and its Supplementary Information File.

## Supplementary Information

Supplementary Information.
