# Supplementary material for: Annexin B12 Trimer Formation is Governed by a Network of Protein-Protein and Protein-Lipid Interactions
Source: Sci Rep. 2020 Mar 24;10:5301. doi: 10.1038/s41598-020-62343-x (PMC7093510; doi:10.1038/s41598-020-62343-x)
Supplement: Supplementary file 1 — Supplementary Information. [file 41598_2020_62343_MOESM1_ESM.pdf]

# Annexin B12 Trimer Formation is Governed by a Network of Protein-Protein and Protein-Lipid Interactions

**Meixin Tao[1], J. Mario Isas[1], Ralf Langen*[1]**

1. Department of Neuroscience and Physiology, Department of Biochemistry and Molecular Medicine, Zilkha Neurogenetic Institute, Keck School of Medicine, University of Southern California, Los Angeles, CA, 90033, USA.

Correspondence and requests for materials should be addressed to R.L.
*email: Ralf Langen: langen@usc.edu

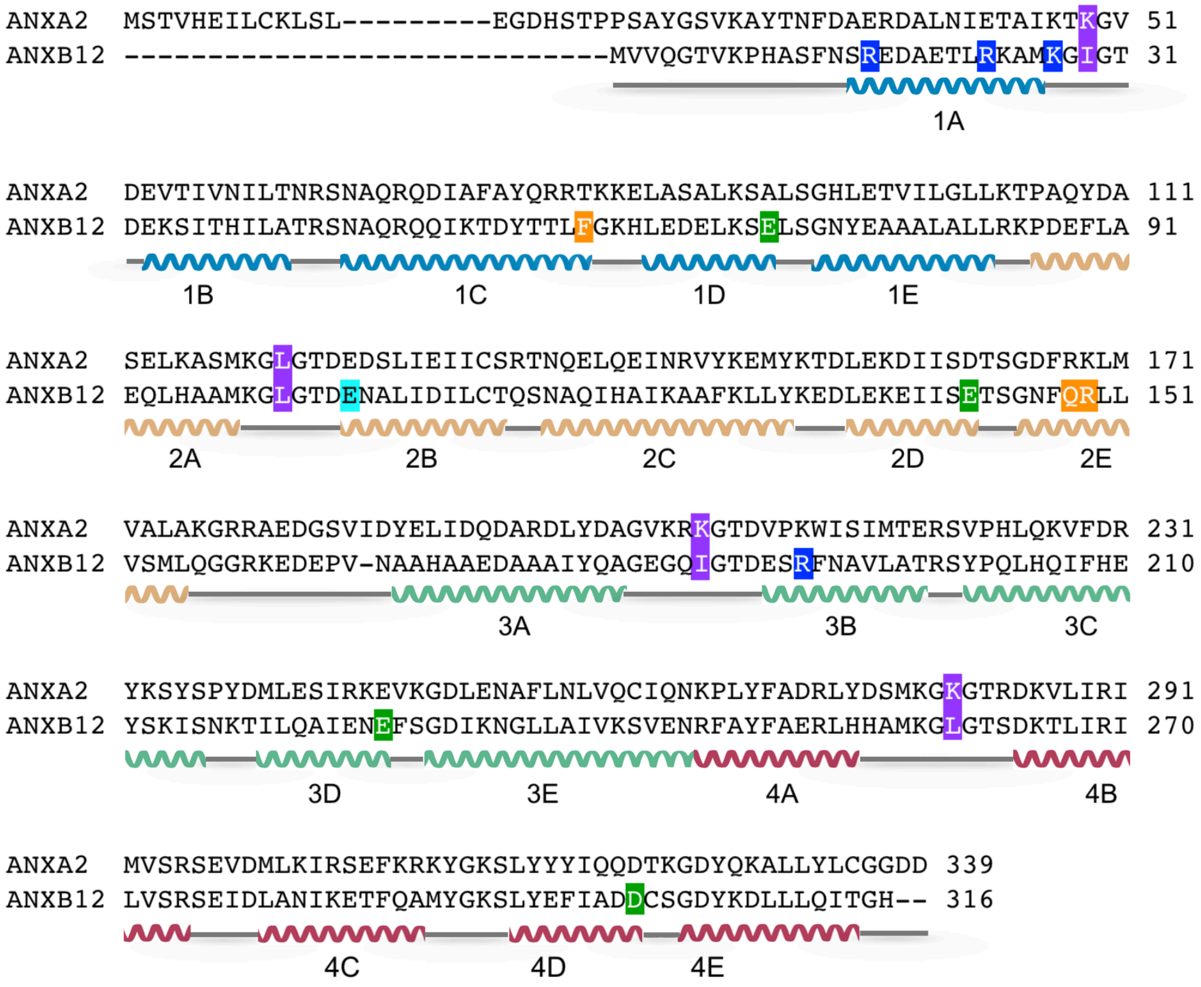


**Supplemental Figure S1.** Sequence alignment of non-trimer forming ANXA2 and trimer-forming ANXB12. The alignment was performed using Clustal Omega. Regions adopting α helical secondary structures are indicated by colored ribbons (color coded the same as in **Fig. 1a**). Each helix is denoted with its respective repeat number (from 1 to 4) followed by helix letter (from A to E). Residues mutated in the present study are highlighted. Positively charged salt bridge residues of ANXB12 are colored in blue. IF-A mutant residues (F59, Q148 and R149) are orange. Mutated Glu/Asp ligands (E70, E142, E226 and D301) of ANXB12 AB $Ca^{2+}$-binding sites are green. The E105 ligand of repeat 2 AB' $Ca^{2+}$-binding site is cyan. Residues at the tip of the AB loops (I29, L101, I185 and L260) are purple. All of these residues are hydrophobic in ANXB12, but three of the equivalent residues in ANXA2 are lysines.

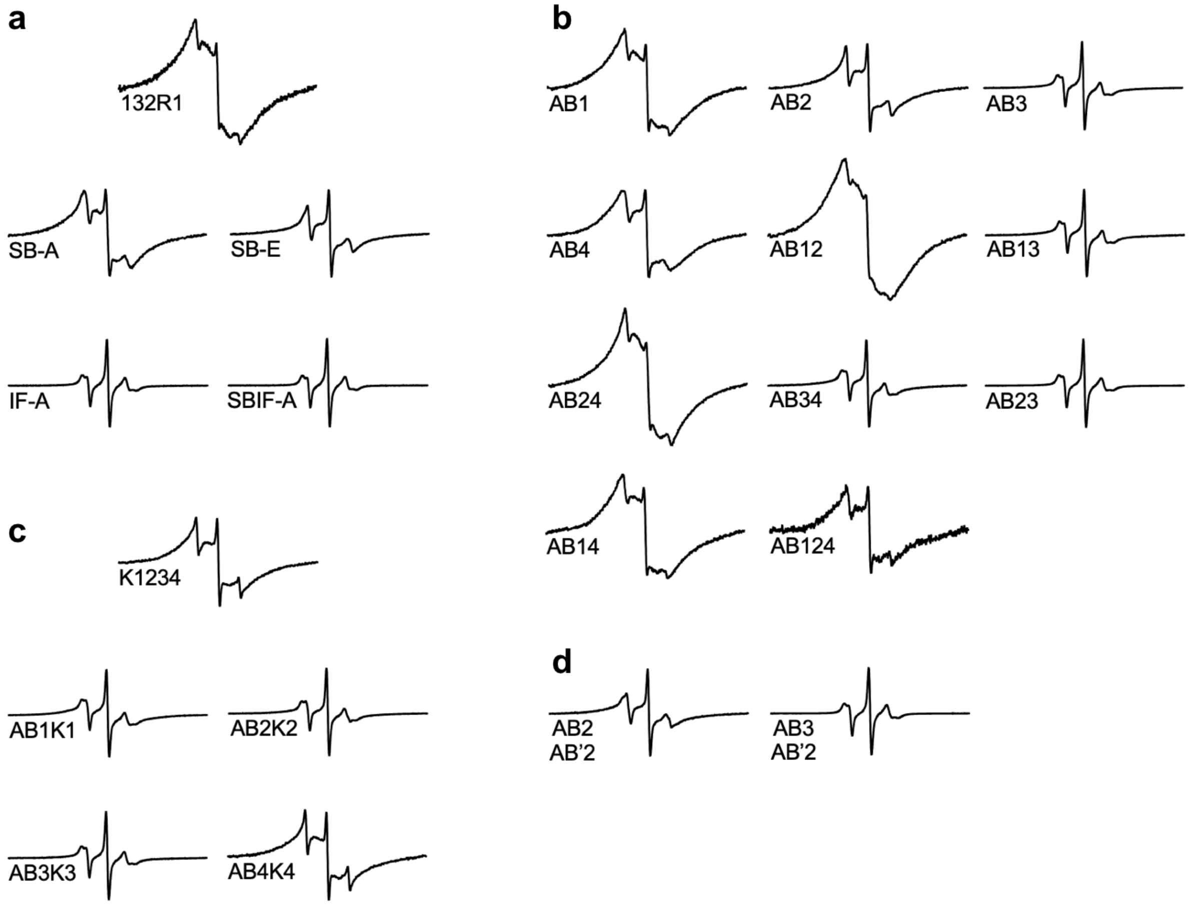


**Supplemental Figure S2. EPR spectra from Fig. 3 shown at same amplitude**. The EPR spectra in Fig. 3 were shown normalized to the same number of spins, which emphasizes the amplitude differences caused by varying amounts of trimer formation. To better illustrate the line shapes of the individual spectra, all spectra are shown here at the same central line amplitude.

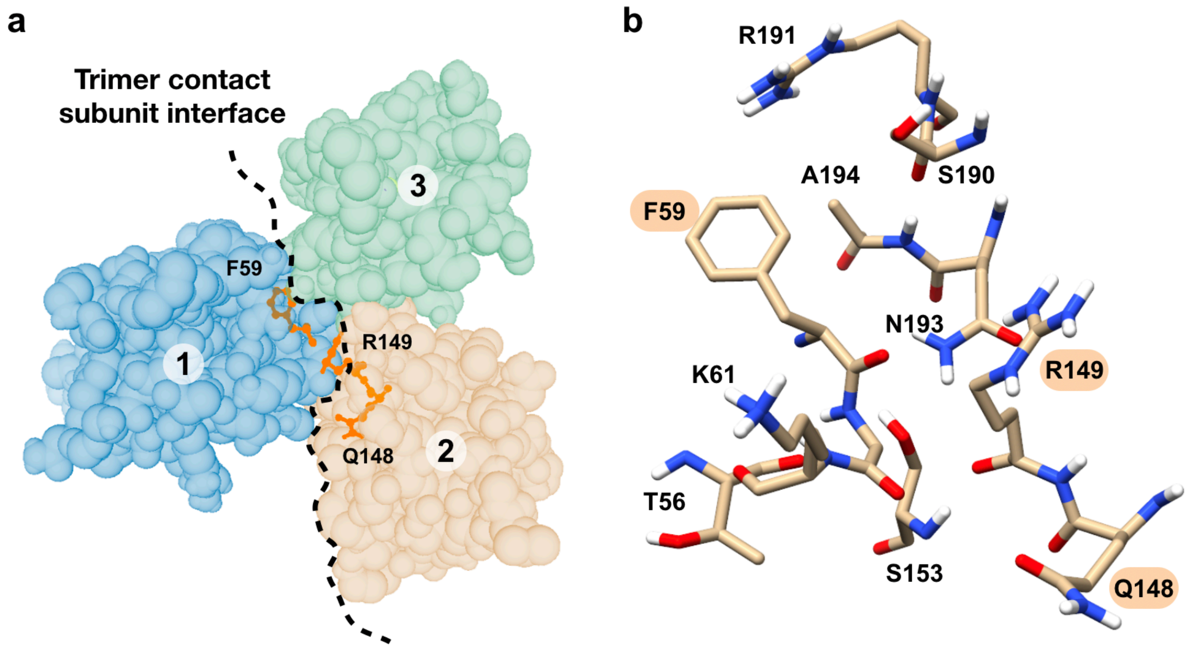


**Supplemental Figure S3. Protein-protein contact surface disrupted by IF-A mutations. (a)** The trimer contact surface is composed of repeat 1 from one subunit and repeats 2/3 from the neighboring subunit. IF-A mutant residues are highlighted in orange. F59 is located in repeat 1 while as Q148/R149 are located in repeat 2. **(b)** Stick representations showing backbones and sidechains of amino acids in contact with IF-A residues according to the crystal structure (PDB code: 1aei).

a

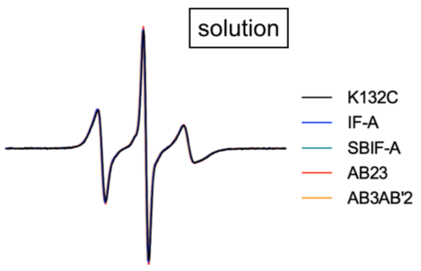


b

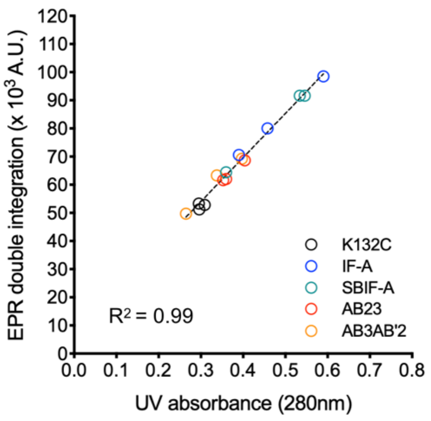


c

| | Repeat # | UV absorbance (280nm) | EPR double integration (x $10^3$ A.U.) | Relative labelling efficiency (%) | Average relative labelling efficiency (%) | Standard deviation |
|---|---|---|---|---|---|---|
| IF-A | 1 | 0.390 | 70.6 | 103.5 | 99.7 | 4.0 |
| | 2 | 0.590 | 98.5 | 95.5 | | |
| | 3 | 0.458 | 80.0 | 100.0 | | |
| SBIF-A | 1 | 0.534 | 91.6 | 98.2 | 98.8 | 3.2 |
| | 2 | 0.546 | 91.6 | 95.9 | | |
| | 3 | 0.360 | 64.4 | 102.3 | | |
| AB23 | 1 | 0.360 | 62.0 | 98.6 | 98.7 | 1.3 |
| | 2 | 0.404 | 68.7 | 97.3 | | |
| | 3 | 0.353 | 61.6 | 100.0 | | |
| AB3AB'2 | 1 | 0.397 | 69.2 | 99.8 | 104.9 | 4.3 |
| | 2 | 0.265 | 49.7 | 107.4 | | |
| | 3 | 0.338 | 63.3 | 107.3 | | |

d

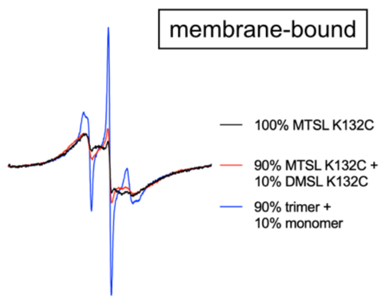


**Supplemental Figure S4. Verification of labelling efficiency for the top four mutants most significantly disrupted in trimer formation. (a)** Solution EPR spectra for reference mutant K132C and the top four mutants most significantly disrupted in trimer formation (IF-A, SBIF-A, AB23 and AB3AB'2) were measured in buffer (20 mM HEPES, 100 mM NaCl, pH 7.4). Their nearly perfect spectral overlap is consistent with an identical local environment. **(b)** To evaluate labelling efficiencies for these four mutants, we estimated the relative protein and spin label concentrations. The former is proportional to 280 nm UV absorbance while the latter is proportional to the double integration of the EPR signal. The two parameters were measured in triplicates and plotted with y-axis showing EPR double integration and x-axis showing UV absorbance at 280 nm. Data fitting shows highly linear relationship ($R^2$ = 0.99). No significant underlabelling relative to the reference mutant K132C was detected, as in all cases the labelling was directly linearly related with protein concentration. Data analysis was performed in GraphPad Prism 8.0. **(c)** The ratios of the two parameters shown in (b) were used to estimate relative label efficiency. Triplicate measurement values of reference mutant K132C were averaged and the ratio was set to be 100% label efficiency. This was done based on the essentially complete spin coupling observed for the spectrum of spin-labelled K132C recorded in its membrane-bound state. Relative labelling efficiencies for the four mutants is then derived from the same ratio in triplicate repeats as shown in the table. (**d**) EPR spectra of membrane-bound ANXB12 K132C mutant (black line) were measured as described in **Fig. 2C**. When replacing 10% MTSL-labelled protein with 10% diamagnetically-labelled protein (DMSL K132C), a small but detectable change in line shape was observed (red line). When we overlay the two spectra with a composite spectrum from 90% membrane-bound trimer and 10% membrane-bound monomer (blue line), the latter has much higher central line amplitude. Thus, underlabelling affects the spectra in a way that is non-linear with respect to apparent monomer concentration. In fact, 10% underlabelling has the same spectral effect as ~2% monomer formation. All spectra were normalized to the same number of spins.
